# Subacute Inhalation Exposure of Mice to Ozone Induces Damage to Various Organs

**DOI:** 10.3390/toxics13060468

**Published:** 2025-05-31

**Authors:** Peiwen Wang, Yuan Lu, Kuikui Lu, Daxiao Xie, Min Ling, Luoding Lu, Weiyong Chen, Yu Wu, Qizhan Liu, Qian Bian, Tian Xiao

**Affiliations:** 1 Laboratory of Modern Environmental Toxicology, Environment and Health Research Division, Public Health School and Health Research Centre, Department of Public Health and Preventive Medicine, Wuxi School of Medicine, Jiangnan University, Wuxi 214122, China; peiwenwang@jiangnan.edu.cn (P.W.); wuyu@jiangnan.edu.cn (Y.W.); 2 Center for Global Health, The Key Laboratory of Modern Toxicology, Ministry of Education, School of Public Health, Suzhou Institute of Public Health, Gusu School, Nanjing Medical University, Nanjing 211166, China; xiedaxiao1997@stu.njmu.edu.cn (D.X.); 2022110424@stu.njmu.edu.cn (W.C.); qzliu@njmu.edu.cn (Q.L.); 3 Institute of Toxicology and Risk Assessment, Jiangsu Province Center of Disease Control and Prevention, Nanjing 210009, China; 32620241151012@stu.xmu.edu.cn (Y.L.); lukk@jscdc.cn (K.L.); lingmin@jscdc.cn (M.L.); luluoding@jscdc.cn (L.L.); 4 School of Public Health, Xiamen University, Xiamen 361005, China

**Keywords:** ozone pollution, inhalation exposure, multiple organ injury, inflammation and oxidative stress, abnormal glucose and lipid metabolism

## Abstract

Ambient ozone (O_3_) pollution, which has become a global problem, is associated with damage to various biological systems, as determined by many studies. However, there is limited experimental evidence regarding the systemic damage induced by O_3_ exposure, and there are few associated studies on mice. In the present investigation, we constructed a subacute C57BL/6J female mouse model involving exposure to 0, 0.5, 1, or 2 ppm O_3_ for 28 days (3 h/day). Body weights, pulmonary function, hematology, serum biochemistry, inflammatory factors, and injuries to various organs were assessed for O_3_-exposed mice. After O_3_ exposure, especially to 2 ppm O_3_, mice showed a loss of body weight, abnormal glucose and lipid metabolism, respiratory and nervous system injuries, an inflammatory response, and pathological changes, which supported the data reported for epidemiology studies. In addition, the IL-6 levels in bronchoalveolar lavage fluid (BALF), the lungs, the livers, the kidneys, and the brains were increased, which indicated that IL-6 was associated with the damage to various organs induced by O_3_ exposure. The present report highlights the pathological injury to various organs and provides a basis for further studies of the molecular mechanisms associated with O_3_ exposure.

## 1. Introduction

With the rapid development of urbanization and industrialization, air pollution has become a serious problem that threatens the health of the global population, a challenge that cannot be ignored [1]. Air pollutants, including particulate matter (e.g., PM_2.5_, PM_10_), volatile organic compounds (VOCs), sulfur dioxide (SO_2_), nitrogen oxides (NO_X_), carbon monoxide (CO), ozone (O_3_), and heavy metals [2], mainly come from human production and human activities (such as industrial emissions, transportation, agricultural activities, energy consumption, and urban dust) and natural hazards (such as volcanic eruptions and wildfires) [3]. According to a report from the World Health Organization (WHO), 99% of people live in areas where the air quality is above the air quality guidelines of WHO, and outdoor pollution contributes to approximately 4.2 million premature deaths worldwide [4]. Thus, studying the health effects induced by air pollution can lead to the development of rules for the prevention of air pollution.

In China, O_3_ presents an air pollution problem greater than that for PM_2.5_ due to clean-air actions implemented since 2013 [5]. High concentrations of tropospheric O_3_ pollutants are harmful to human health [6]. With the action of high temperatures and strong light radiation, the NO_X_ and VOCs produced by vehicle exhaust emissions, fossil fuel combustion, and industrial production processes generate O_3_ through photochemical reactions [7]. In a modeling study on 12,946 cities worldwide, O_3_-attributable mortality and the average population-weighted mean O_3_ concentration increased by 46% and 11%, respectively, from 2000 to 2019. The number of cities with O_3_ concentrations over 60 μg/m^3^ (the WHO peak season standard) increased from 89% in 2000 to 96% in 2019 [8]. Thus, O_3_ exposure is increasing worldwide, contributing to attributable mortality.

O_3_, a highly reactive oxidant gas, is associated with damage to various organs and body systems. A prospective cohort study of lung function among middle-aged European adults shows that long-term exposure to high O_3_ concentrations is associated with a faster decline in function [9]. O_3_ exposure is associated with airway inflammation, emphysema, chronic obstructive pulmonary disease (COPD), airway remodeling, pulmonary fibrosis, and other lung diseases [10,11,12]. Further, O_3_ exposure affects respiratory health through molecular and cellular perturbations in the respiratory tract [13]. Evidence from national cohort studies in China shows that long-term O_3_ exposure contributes to elevated mortality risks of cardiovascular diseases (CVDs), especially ischemic heart diseases in a middle-income setting [14,15,16]. After inhalation into the respiratory tract, O_3_ reduces the peripheral immune response and is connected with an increased risk of Alzheimer’s disease [17], and chronic ambient O_3_ exposure increases the Aβ plaque load and enhances autism-like symptoms [17,18]. In the current study, there is evidence of the damage to the respiratory system *in vivo* and *in vitro* [19,20], the cardiovascular system *in vivo* and *in vitro* [21,22], the liver *in vivo* [23], and the nervous system *in vivo* [24] induced by O_3_ exposure. However, there is no experimental evidence either *in vivo* or *in vitro* of any damage to the spleen caused by exposure to ambient O_3_.

Based on these reports, the present study intended to construct a 28-day O_3_ subacute exposure mouse model, to collect tissue samples, and to observe the pathological changes in various organs. The findings give us a better understanding of the health risks associated with ambient O_3_ exposure and lay the foundation for further research on the specific molecular mechanisms of O_3_-induced diseases.

## 2. Methods and Materials

### 2.1. Animal Models

For female mice, air pollution, including O_3_ exposure, has severe effects on lung tissue inflammation and damage [25]. C57BL/6J female mice (6–8 weeks old, SPF grade), provided by the Laboratory Animal Center of Nanjing Medical University, were randomly divided into groups subjected to 0, 0.5, 1, or 2 ppm O_3_ exposure (*n* = 12). The mice were exposed to these concentrations for 28 days (3 h/day). The device for monitoring O_3_ generation (CHUAVG, Guangzhou, China) connected to the whole-body exposure system for rodents (Beijing Huironghe Technology Co., Ltd., Beijing, China) constituted an O_3_ exposure chamber. The detector monitored the O_3_ concentrations in real time at the respiratory height of mice under O_3_ exposure. Parameters such as temperature, humidity, and the concentrations of O_2_ and CO_2_ were monitored and recorded in real time by the exposure system. After exposed to O_3_ for 28 days, the mice were anesthetized by an intraperitoneal injection of pentobarbital sodium, and then they were sacrificed, dissected, and sampled. All the mice were housed in an SPF environment and fed with a standard diet at 22 °C with 50–60% humidity and an equal light–dark cycle. If the mice showed poor health conditions such as emaciation during this experiment, they were euthanized immediately by carbon dioxide inhalation and reported on. The detailed experimental plans of each group of mice are shown in Table S1. And the animal experiments were approved by the Animal Care and Use Committee of Jiangsu Provincial Center for Disease Control and Prevention (JSJK/JL-161).

### 2.2. Basis for Doses of O_3_

Based on the global pollution situation and limit values of O_3_, we determined the O_3_ exposure dose for mice. The Ambient Air Quality Standard of China (GB 3095-2012) stipulates that the daily maximum 8 h average O_3_ (MDA-8h O_3_) was about 0.08 ppm after conversion according to the secondary standard of 160 μg/m^3^ (1 ppm = 2.14 mg/m^3^) [26]. In 2022, the 90th percentile of MDA-8h O_3_ in 339 cities in China was 90–194 μg/m^3^, which is about 0.1 ppm after conversion [27]. Further, considering the difference in volume and lung surface area between rodents and humans, to achieve the same deposition and inflammatory response as that of humans, 4–5 times the dose was required to derive the exposure dose of rodents [28,29,30]. Thus, we set the minimum dose of O_3_ at 0.5 ppm, and the doses of O_3_ exposure were set at 0, 0.5, 1, and 2 ppm with an equal proportional gradient.

### 2.3. Analysis of Mouse Pulmonary Function

At the end of the O_3_ exposure, the pulmonary function of mice was measured by a whole-body system (Buxco Electronics Ltd., Wilmington, NI, USA) (*n* = 12). The mice were placed in a tracing cavity and acclimated for 10 min. Then, 0, 12.5, 25, or 50 mg/mL acetylcholine (Ach) was added to the cavity and nebulized for 2 min with a period of responding for 3 min and recovering for 2 min. The relevant pulmonary function parameters, including respiratory frequency (F), mid-expiratory flow rate (EF50), and airway stenosis index (enhanced expiratory pause, Penh), were recorded by FinePoint software (software version, 2.3.1.14) (Buxco Electronics Ltd., USA).

### 2.4. Extraction of BALF

After anesthetizing the mice with pentobarbital sodium, the trachea and thoracic cavity of the mice were fully exposed, with one bronchial tube being ligated. A small portion of the tracheal wall was gently elevated, and a 1 mL syringe loaded with 0.5 mL of physiological saline was inserted into the trachea and secured. The syringe was gently depressed to observe the dilation of the other lung. After allowing it to dwell for 30 s, the BALF was slowly aspirated. The collected BALF (*n* = 6) was centrifuged at 1000 r/min for 10 min to remove cells and then stored at −80 °C for future use.

### 2.5. Hematology and Serum Biochemistry

At the end of the O_3_ exposure and pulmonary function assessments, the mice were anesthetized by the intraperitoneal administration of pentobarbital (Entoval^®^, Hallym Pharm. Co., Ltd., Seoul, Republic of Korea), and whole blood was collected. The hematology parameters in the blood (*n* = 6) were measured by an ADVIA 2120 hematology system (SIMENS, Munich, Germany). Other blood samples (*n* = 6), collected from the abdominal aorta, were used to collect serum in heparinized vacutainers for analysis by a biochemical blood analyzer (Olympus, Tokyo, Japan).

### 2.6. Histological Analysis

After exposure to 0, 0.5, 1, or 2 ppm O_3_, the mice were sacrificed, and tissues were harvested (*n* = 6). Half of the blood, brain, heart, trachea, lung, liver, kidney, pancreas, and spleen tissues were fixed in paraformaldehyde, embedded in paraffin, and then made into specimens. The specimens were exposed to hematoxylin–eosin (H&E) for histopathological evaluation. The staining images were observed by a CaseViewer 2.4 (3DHISTECH Ltd., Budapest, Hungary).

### 2.7. Enzyme-Linked Immunosorbent Assay (ELISA)

Frozen tissue samples were retrieved and thawed under controlled conditions (4 °C for 15 min). Surface moisture was removed by gentle blotting with sterile filter paper. A total of 0.1 g of tissue was precisely weighed before being flash-frozen in liquid nitrogen for cellular structure preservation. A total of 0.9 mL of ice-cold sterile saline was added and mechanically homogenized at 30 Hz for 2 min. The resultant homogenate was centrifuged at 3000 r/min for 20 min at 4 °C to obtain a clarified supernatant for subsequent analyses according to the instructions of the manufacturer. The levels of interleukin-6 (IL-6), interleukin-8 (IL-8), tumor necrosis factor-alpha (TNF-α), and high-mobility group protein (HMGB1) were measured with Mouse IL-6 ELISA Kits (CSB-E04639m, CUSABIO, Wuhan, China); Mouse IL-8 ELISA Kits (YFXEM00011, Yfxbio Biotech. Co., Ltd., Nanjing, China); Mouse TNF-α ELISA Kits (CSB-E04741m, CUSABIO, Wuhan, China); and Mouse HMGB1 ELISA Kits (CSB-E08225m, CUSABIO, Wuhan, China), respectively. Finally, the optical density of each well was determined using a SpectraMax Paradigm (Molecular Devices, Sunnyvale, CA, USA) set to 450 nm.

### 2.8. Statistical Analysis

All experiments were performed in triplicate, and the data of each sample are represented by the mean of three repeated experiments. The data are reported as means ± standard deviation (SD). For comparisons of means among multiple groups, statistical analysis was performed by using SPSS14.0 for a one-way analysis of variance (ANOVA). Inter-group comparisons were accomplished by calculating multiple-range least significant differences. Graphs were prepared with GraphPad Prism 9 software. *p* values *<* 0.05 were considered to be statistically significant.

## 3. Results

### 3.1. The Body Weights, Serum Biochemistry, and Hematology Parameters of Mice Are Affected by Subacute Exposure to O_3_

**Figure 1:**
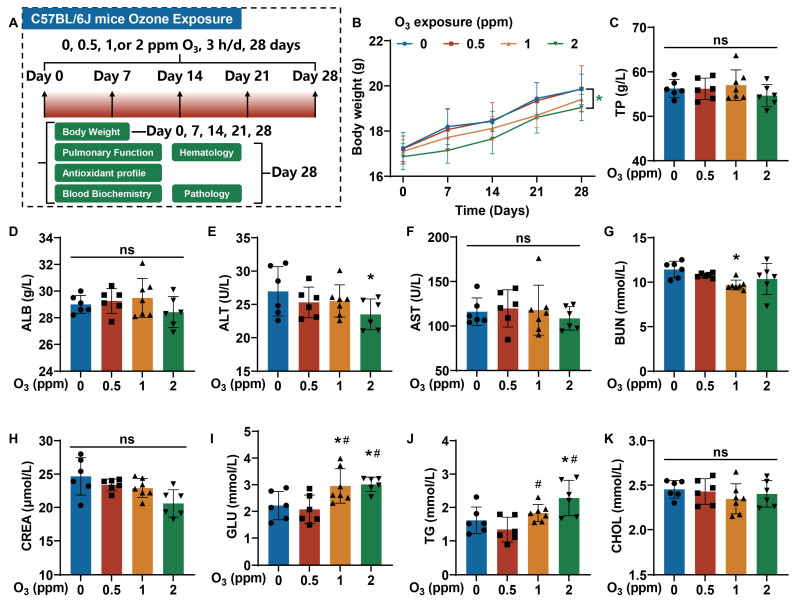
Changes in the body weights and serum biochemistry parameters of mice exposed to O_3_ for 28 days. Female C57BL/6J mice, aged 6–8 weeks, were exposed to 0, 0.5, 1, or 2 ppm O_3_ for 28 days (3 h/d). On the 7th, 14th, 21st, and 28th days, the body weights of mice were measured. After 28 days of O_3_ exposure, the mice were sacrificed, and their blood and organs were harvested. (**A**) A schematic illustration of the construction of the C57BL/6J mouse model with O_3_ exposure. (**B**) Changes in the body weights of mice. * Green, *p* < 0.05, 2 ppm vs. 0 ppm O_3_ exposure groups. (**C**) The blood concentrations of TP, (**D**) ALB, (**E**) ALT, (**F**) AST, (**G**) BUN, (**H**) CREA, (**I**) GLU, (**J**) TG, and (**K**) CHOL. The data are presented as means ± SD, *n* = 6. *, *p* < 0.05, compared with the 0 ppm O_3_ exposure group. #, *p* < 0.05, compared with the 0.5 ppm O_3_ exposure group. ns, non-significant.

Based on the health effects of O_3_, a mouse model of O_3_ exposure was constructed. C57BL/6J mice were exposed to 0, 0.5, 1, or 2 ppm O_3_ for 28 days (3 h/d). On days 0, 7, 14, 21, and 28, the body weights of mice were measured. After their pulmonary function was assessed on day 28, the mice were sacrificed, and their tissues were harvested. Body weight-, pulmonary function-, antioxidant profile-, blood biochemistry-, hematology-, and pathology-related parameters were examined (Figure 1A). Compared with the 0 ppm O_3_ group, the body weights of mice in the 2 ppm O_3_ group were lower (Figure 1B). In blood biochemistry tests, we measured indicators of liver function (total protein (TP), albumin (ALB), alanine aminotransferase (ALT), and aspartate aminotransferase (AST)); kidney function (blood urea nitrogen (BUN) and creatinine (CREA)); and glycolipid metabolism (glucose (GLU), triglycerides (TGs), and cholesterol (CHOL)). Changes in TP (Figure 1C), ALB (Figure 1D), AST (Figure 1F), CREA (Figure 1H), and CHOL (Figure 1K) concentrations in mouse blood were not statistically significant. The concentrations of ALT were lower in the blood of mice exposed to 2 ppm O_3_ compared to the 0 ppm O_3_ group (Figure 1E), but there was no clinical significance. The BUN levels of mice exposed to 1 ppm O_3_ were lower than those for the 0 ppm O_3_ group (Figure 1G). The blood concentrations of GLU (Figure 1I) and TG (Figure 1J) were higher in mice exposed to O_3_ compared to controls. The values are shown in Table S2.

Further, blood biochemistry parameters were measured (Table S3). The values of white blood cells (WBCs), the mean corpuscular volume (MCV), red cell distribution width standard deviation (RDW-SD), platelet distribution width (PDW), neutrophils (NEUT), lymphocytes (LYMPH), monocytes (MONO), and eosinophils (EO) were changed after exposure to various levels of O_3_ (Table S3). The parameters for the 1 ppm group were lower than those for the 0.5 and 2 ppm O_3_ groups. The large differences between individual animals may have contributed to this observation. Therefore, the loss of body weight, abnormal glucose and lipid metabolism, and the abnormal hematology parameters in mice were related to subacute exposure to O_3_ for 28 days.

### 3.2. Respiratory Tract Damage Is Induced in Mice by Subacute Exposure to O_3_

**Figure 2:**
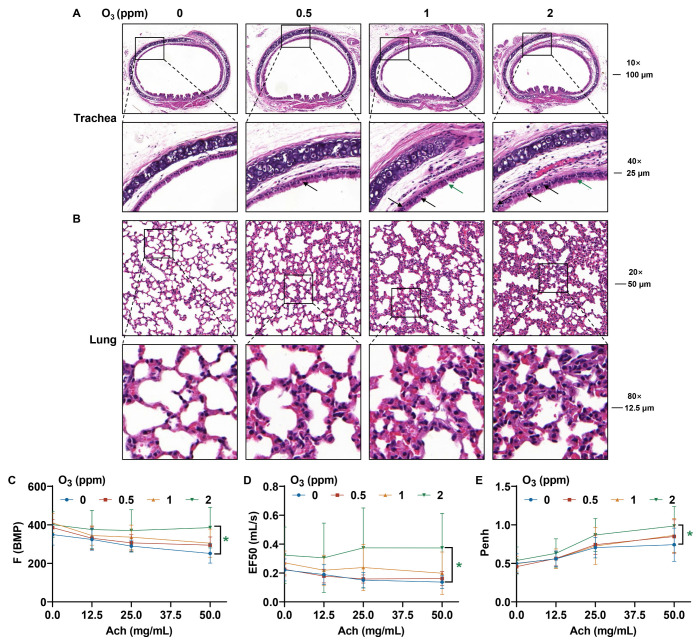
Pathological changes in the trachea and lung tissues and changes in pulmonary function in O_3_-exposed mice. (**A**) Representative H&E staining images of trachea (Scale bar, 10×, 100 μm; 40×, 25 μm). The black arrows indicate bronchial mucosal epithelial goblet cells. The green arrow shows the loss of cilia. (**B**) Representative H&E staining images of lung tissues (Scale bar, 20×, 50 μm; 80×, 12.5 μm). (**C**) Changes in the respiratory function parameters, including the F, (**D**) EF50, and (**E**) Penh values of O_3_-exposed mice. The data are reported as means ± SD, *n* = 12. * Green, *p* < 0.05, 2 ppm vs. 0 ppm O_3_ exposure groups.

**Figure 3:**
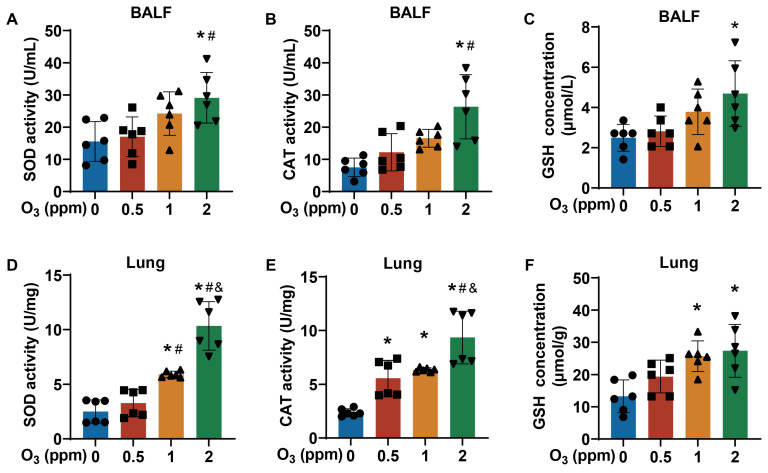
Oxidative stress status of BALF and lungs of O_3_-exposed mice. (**A**) Levels of SOD activity, (**B**) CAT activity, and (**C**) GSH concentration in BALF of O_3_-exposed mice. (**D**) Levels of SOD activity, (**E**) CAT activity, and (**F**) GSH concentration in lung tissues of O_3_-exposed mice. Data are reported as means ± SD, *n* = 6. *, *p* < 0.05, compared with 0 ppm O_3_ exposure group. #, *p* < 0.05, compared with 0.5 ppm O_3_ exposure group. &, *p* < 0.05, compared with 0.5 ppm O_3_ exposure group.

Lungs are a direct target for atmospheric O_3_ exposure [31,32]. From the H&E staining images of trachea tissues, compared to the control groups, for O_3_-exposed mice, there were elevated numbers of bronchial mucosal epithelial goblet cells (black arrow) and a loss of cilia (green arrow) (Figure 2A). After subacute exposure to O_3_, the normal alveolar structure in mouse lung tissues was broken, the alveolar walls were slightly thickened, and inflammatory cells were present (Figure 2B). After stimulation with Ach, compared with the control group, the respiratory frequency (F), mid-expiratory flow rate (EF50), and airway stenosis index (enhanced expiratory pause, Penh) were elevated in the group exposed to 2 ppm O_3_ (Figure 2C–E). In addition, oxidative stress-related parameters, including superoxide dismutase (SOD), catalase (CAT), and glutathione (GSH), were detected in the bronchoalveolar lavage fluid (BALF) and lung tissues of mice exposed to different doses of O_3_. The SOD activity (Figure 3A) and CAT activity (Figure 3B) levels in BALF were higher in mice exposed to 2 ppm O_3_ compared to the 0 and 0.5 ppm O_3_ groups. Compared to controls, the GSH concentration in BALF was significantly increased in 2 ppm O_3_-exposed mice (Figure 3C). In lung tissues, with an increase in O_3_ doses, the SOD activity (Figure 3D) and CAT activity (Figure 3E) levels were increased in a dose–effect manner. And the GSH concentration in lung tissues was significantly increased in the 1 and 2 ppm O_3_-exposed mice compared to controls (Figure 3F). The results indicated that, for mice, O_3_ exposure caused damage to the trachea and lungs, pulmonary dysfunction, and oxidative stress in BALF and the lungs.

### 3.3. The Pathological Injury to Various Organs Is Involved in O_3_ Exposure

**Figure 4:**
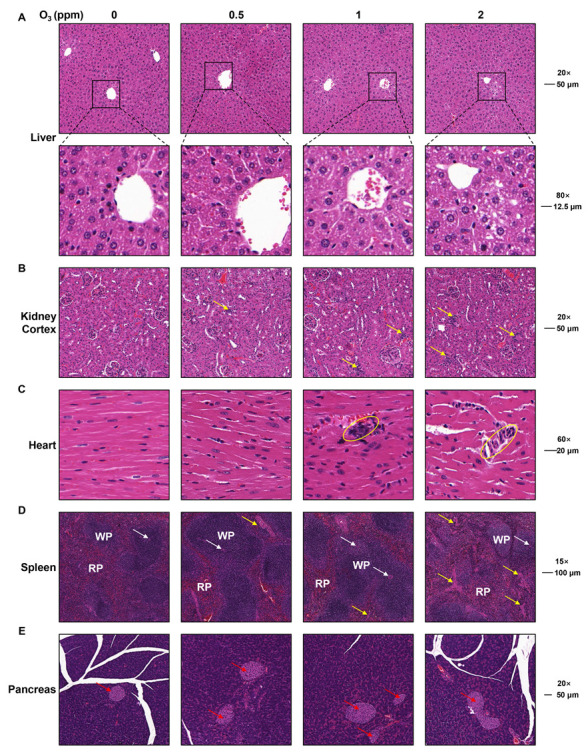
Pathological changes in heart, spleen, pancreas, liver, and kidneys in O_3_-exposed mice. (**A**) Representative H&E staining images of liver (Scale bar, 20×, 50 μm; 80×, 12.5 μm). (**B**) Representative H&E staining images of kidney cortex (Scale bar, 20×, 50 μm). Yellow arrows show inflammatory infiltration. (**C**) Representative H&E staining images of heart (Scale bar, 60×, 20 μm). Yellow circles show inflammatory infiltration. (**D**) Representative H&E staining images of spleen (Scale bar, 15×, 100 μm). WP represents white pulp. RP represents red pulp. Yellow arrows indicate spleen trabecula. White arrows indicate splenic arterioles. (**E**) Representative H&E staining images of pancreas (Scale bar, 20×, 50 μm). Red arrows indicate pancreatic islets.

The organ injuries induced by O_3_ exposure were assessed by H&E staining. In the livers of mice exposed to O_3_, hepatocytes were disordered, and the intercellular space was enlarged (Figure 4A). Compared with the control groups, for O_3_-exposed mice, there was an infiltration of inflammatory cells into the kidney cortex (Figure 4B, yellow arrows) and heart (Figure 4C, yellow circles). In the spleens of mice in the 2 ppm O_3_ group, the trabeculae (yellow arrows) were increased (Figure 4D. WP, white pulp; RP, red pulp; white arrows, splenic arterioles). However, there was no significant difference in the pancreases of mice exposed to O_3_ (Figure 4E). Thus, these data indicate that the O_3_ exposure of mice for 28 days induces injuries to various organs.

### 3.4. The Degree of Inflammatory Response Is Associated with O_3_ Exposure

**Figure 5:**
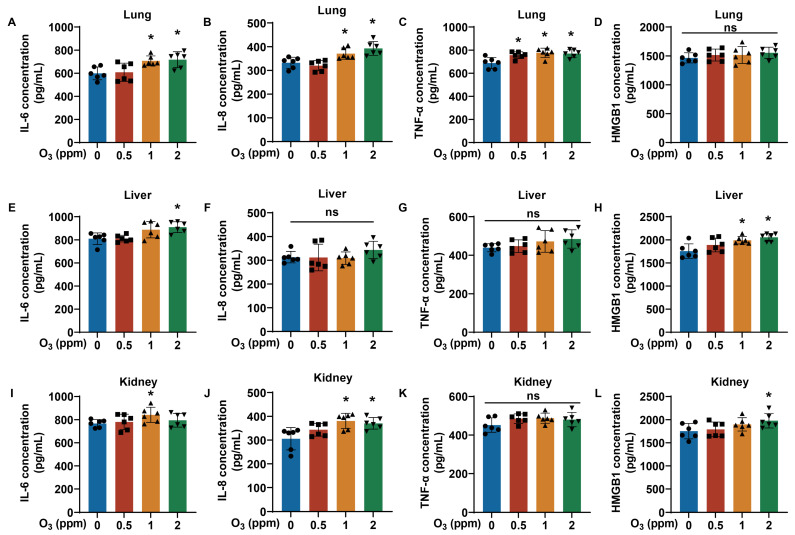
Changes in inflammatory factor levels in lung, liver, and kidney tissues of O_3_-exposed mice. (**A**) Levels of IL-6, (**B**) IL-8, (**C**) TNF-α, and (**D**) HMGB1 in lung tissues of O_3_-exposed mice. (**E**) Levels of IL-6, (**F**) IL-8, (**G**) TNF-α, and (**H**) HMGB1 in liver tissues of O_3_-exposed mice. (**I**) Levels of IL-6, (**J**) IL-8, (**K**) TNF-α, and (**L**) HMGB1 in kidney tissues of O_3_-exposed mice. Data are reported as means ± SD, *n* = 6. *, *p* < 0.05, compared with 0 ppm O_3_ exposure group. ns, non-significant.

It is reported that the occurrence of oxidative stress can cause an immune cascade reaction, resulting in the release of IL-6, IL-8, TNF-α, and so on [33]. Therefore, we further examined the levels of inflammatory factors in the tissues of O_3_-exposed mice. For lung, liver, and kidney tissue, we measured the levels of inflammatory cytokines, including IL-6, IL-8, TNF-α, and HMGB1. Elevated levels of IL-6, IL-8, and TNF-α were present in lung tissues of mice after exposure to O_3_ for 28 days (Figure 5A–C). However, in the lung tissues of O_3_-exposed mice, the levels of HMGB1 were not changed significantly (Figure 5D). In liver tissues, the IL-6 levels in the 2 ppm O_3_ group and the HMGB1 levels in the 1 and 2 ppm O_3_ groups were elevated, but the levels of IL-8 and TNF-α were unchanged (Figure 5E–H). Moreover, compared with the 0 ppm O_3_ group, the IL-6 levels in the 1 ppm O_3_ group, the IL-8 levels in the 1 and 2 ppm O_3_ groups and the HMGB1 levels in the 2 ppm O_3_ group were upregulated, but this was not observed for the kidney levels of TNF-α in mice exposed to O_3_ (Figure 5I–L). Thus, the inflammatory responses that especially increased IL-6 levels in the lungs, liver, and kidneys are induced by O_3_ exposure for 28 days. In addition, the elevated levels of IL-6 in the liver and kidneys may be directly or indirectly (potentially secreted from pulmonary effects) induced by O_3_ exposure.

### 3.5. Pathological Damage and Inflammatory Responses in Brain Are Related to O_3_ Exposure

**Figure 6:**
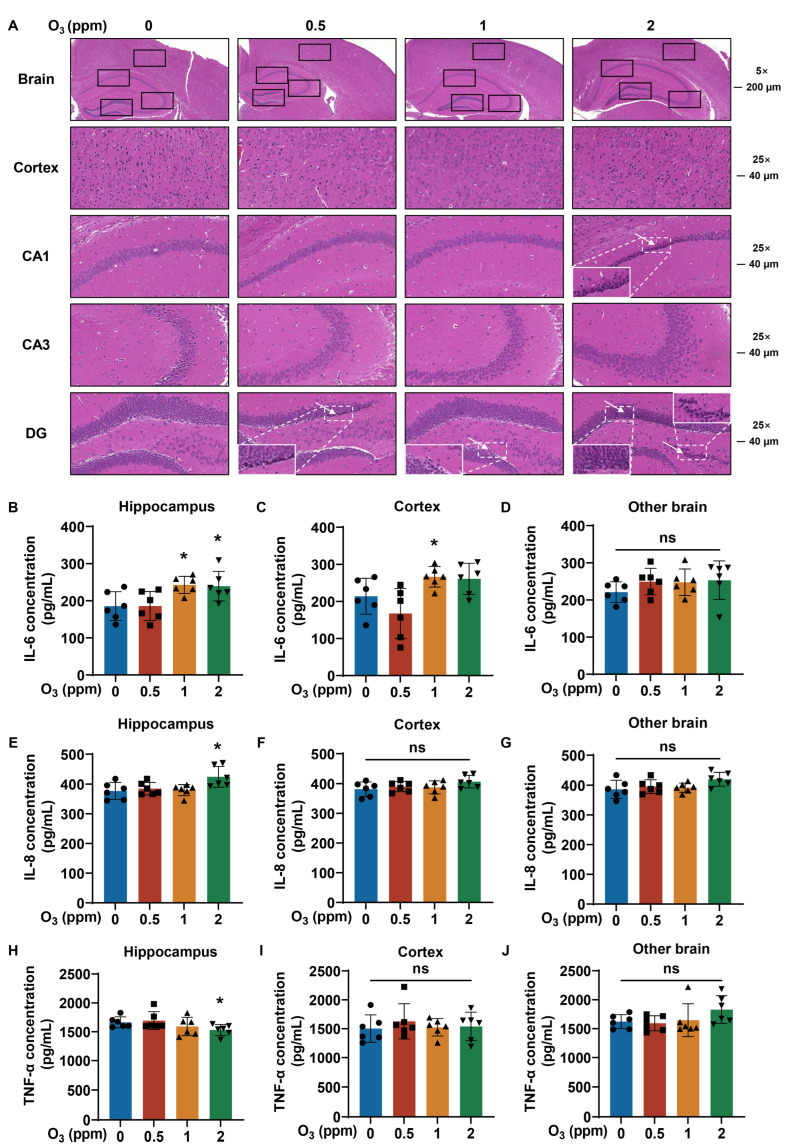
Pathological inflammatory changes in brain tissues of O_3_-exposed mice. (**A**) Representative H&E staining images of brain (Scale bar, 5×, 200 μm; 25×, 40 μm). The hippocampus, cortex, CA1, CA3 and DG of brain were marked with black boxes. White arrows show shrunken nuclei of neurons, and corresponding area image is magnified. (**B**) Levels of IL-6, (**E**) IL-8, and (**H**) TNF-α in hippocampus of brain tissues of O_3_-exposed mice. (**C**) Levels of IL-6, (**F**) IL-8, and (**I**) TNF-α in brain cortex of O_3_-exposed mice. (**D**) Levels of IL-6, (**G**) IL-8, and (**J**) TNF-α in other brain of O_3_-exposed mice. Data are reported as means ± SD, *n* = 6. *, *p* < 0.05, compared with 0 ppm O_3_ exposure group. ns, non-significant.

Because diseases of the nervous system are related to O_3_ exposure [17], we next investigated injury to the cortex and hippocampus, including the cornu ammonis 1 (CA1), the cornu ammonis 2 (CA2), and the dentate gyrus (DG) of the brains of mice exposed to O_3_. For the brains of mice exposed to O_3_, H&E staining showed the atrophy of neuronal nuclei (white arrows) in CA1 and DG and the disorganized and sparse arrangement of neurons in the cortex, CA3, and DG (Figure 6A). Compared with those in the 0 ppm O_3_ group, IL-6 levels were elevated in the hippocampus in the 1 and 2 ppm O_3_ groups (Figure 6B) and in the cortex of the 1 ppm O_3_ group (Figure 6C); there were no significant changes in IL-6 levels in other brain areas after O_3_ exposure (Figure 6D). In the hippocampus, the IL-8 levels were elevated in the 2 ppm O_3_ group compared to those in the 0 ppm O_3_ exposure group (Figure 6E). However, after O_3_ exposure, there were no significant changes in the cortex (Figure 6F) or other areas (Figure 6G) of the brain. In addition, the levels of TNF-α were lower in the hippocampus of the brains of mice dosed with 2 ppm O_3_ (Figure 6H), but there were no differences in TNF-α levels in the cortex (Figure 6I) or in other areas (Figure 6J) of the brain between groups with different doses of O_3_. Thus, we conclude that the pathological damage and inflammatory responses (especially IL-6) in the brain are induced by O_3_ exposure for 28 days.

## 4. Discussion

O_3_ is a common secondary pollutant in the atmospheric troposphere, and in recent years, O_3_ pollution in China and in the world has become increasingly severe [34,35]. According to the State of Global Air 2020 report, with global warming and the increasing emissions of O_3_ precursors, global ground surface O_3_ concentrations are 30% to 70% higher than they were 100 years ago [36]. In China, the ambient concentrations of PM_2.5_ have been reduced, while ground O_3_ concentrations near the ground surface have increased after the implementation of clean-air actions in 2013 [5]. Although the impacts, including health and economic ones, induced by O_3_ pollution are less severe than those induced by PM_2.5_, they are more difficult to mitigate [37]. Thus, we believe that promoting control policies for air pollution, especially O_3_ pollution, can reduce the harm that O_3_ has on health.

O_3_ is highly oxidizing and induces the accumulation of reactive oxygen species (ROS) after entering lung tissue, which activates several signaling cascades, including oxidative stress, autophagy, and pyroptosis [38,39]. The oxidative stress response activates its relative pathway and plays an important role in the respiratory system injuries induced by ambient O_3_ exposure [31,40,41,42]. For the lung tissues of O_3_-exposed mice, experiments show that there is oxidative stress and inflammation, which may reduce immunity and promote the development of asthma, emphysema, COPD, and pulmonary fibrosis [31,43,44,45]. In our study, we found that the levels of oxidative stress-related indicators, such as SOD, CAT, and GSH, were significantly increased due to the exposure of O_3_ in the lung tissues of mice, which was consistent with the reported studies.

Some evidence shows that O_3_ exposure can lead to liver damage and a blood glucose metabolism disorder in rats [46,47]. Consistently, the serum biochemistry results in our study showed that the GLU and TG concentrations in the blood of mice were elevated after O_3_ exposure, indicating that the blood glucose metabolism disorder was induced by O_3_ exposure. In our research, the values of BUN in serum biochemistry and MCV in hematology were decreased in the blood of mice exposed to O_3_. These changes are possibly associated with iron deficiency and an iron absorption disorder [48]. The present evidence provides ideas for further research on the abnormal iron metabolism and oxidative stress associated with O_3_ exposure and on the molecular mechanisms of how O_3_ exposure affects health.

The adverse effects of increasing O_3_ concentrations on human health have become a serious problem of air pollution in China and globally [49]. For example, a cohort study in the United States showed that ambient O_3_ concentrations were associated with greater increases in the incidence of emphysema and a decline in forced expiratory volume of 1 s per 10 years [50]. Further, by releasing IL-2 and TNF-α, O_3_ induces inflammation, which promotes hypertension, coronary ischemia, and the impairment of autonomic control in the lungs and in the circulation [51]. Epidemiological studies in China show the strong association between long-term exposure to high levels of O_3_ and cause-specific CVD mortality [16]. As shown in a randomized controlled trial, O_3_ causes an increase in the vascular markers of inflammation and decreases in the markers of fibrinolysis [52]. In addition, long-term O_3_ exposure is associated with higher risks of allergic rhinitis, conjunctivitis, eczema, and poor bone development in children [53,54]. Other damage includes liver fibrosis and a blood glucose metabolism disorder in rats [46,47]. We found that high-dose O_3_-exposed mice exhibited respiratory system injury, hepatic interstitial expansion, splenic trabecular hyperplasia, and notable inflammatory cell infiltration in heart and kidney tissues, compared to the control groups.

In the analysis of mouse pulmonary function, Ach was atomized and released slowly into the resting chamber to keep all the mice in a resting state. After the detection of pulmonary function, the mice regained consciousness in about half an hour. Studies have shown that Ach may have an impact on airway resistance and other factors [55,56]. In this investigation, several methodological considerations were implemented to mitigate potential confounding effects: (1) a relatively low inhalational dose was administered to expedite systemic clearance, given the compound’s rapid metabolic turnover; (2) sample collection was strategically deferred until the subsequent day following pulmonary function assessment to allow for the complete elimination of residual Ach; (3) a standardized exposure protocol was applied uniformly across all mice (*n* = 12), ensuring equivalent pharmacological stimulation; and (4) only the levels of lung function indicators under the stimulation of the same concentration of Ach were compared, which to a certain extent controlled the effects caused by Ach. This rigorous experimental design approach effectively controls for both temporal variability in drug metabolism and inter-group variability in Ach exposure, thereby enhancing the interpretative validity of the observed physiological responses. Although we administered Ach, the potential confounding effects attributable to Ach were successfully mitigated through meticulous experimental design and rigorous analytical methods. In our study, we found that O_3_ exposure induced the damage of pulmonary function.

Since O_3_ is a reactive air pollutant that cannot be transferred to the brain to affect the central nervous system [17,57], damage to the brain is possibly induced by O_3_-affected lungs via an organ–organ axis [58]. Some studies prove that O_3_ exposure is associated with autism-like symptoms, neurodegenerative diseases, neuroinflammation, cognitive impairment, a depressive-like response, and Alzheimer’s disease [18,42,59,60,61]. For mice, O_3_ exposure leads to increased Aβ plaque load and augmented dystrophic neurites through an elevated lung proinflammatory response and peripheral HMGB1 levels via a lung–brain axis [17]. Our results indicated that there was an atrophy of neuronal nuclei, a disorganized and sparse arrangement of neurons, and enhanced levels of IL-6 in the cortex and hippocampus of mice exposed to 2 ppm O_3_. Thus, we supposed that the damage to the brain is involved in the hyper-IL-6 levels seen via the lung–brain axis after O_3_ exposure.

In our study, we found increasing levels of IL-6 in the lungs, livers, kidneys, and brains of mice exposed to high doses of O_3_. In addition, for mice with 2 ppm O_3_ exposure, there were disorders in hematology and serum biochemistry, respiratory tract injury, and pathological changes in the livers, kidneys, hearts, spleens, pancreas, and brains, as well as changes in oxidative stress-related parameters in BALF and the lungs. Based on the evidences above, we speculate that O_3_ exposure causes oxidative stress in the lungs and the release of inflammatory factors, especially IL-6, and may lead to various types of organ damage via the lung–organ axis. However, regrettably, our study only focused on observing the phenotypic alterations induced by O_3_ exposure and did not validate the specific underlying mechanisms. Even so, the role of IL-6 in the lung–organ axis can be explored in the future. The results establish a base for studies on molecular mechanisms to elucidate the potential pathways underlying O_3_-mediated extra-pulmonary injuries.

## 5. Conclusions

Mice exposed to O_3_ for 28 days demonstrated oxidative stress, abnormal glucose and lipid metabolism, respiratory system injury, an inflammatory response, and injury to various organ and nervous systems. This study described the injuries to various organs caused in mice by subacute O_3_ exposure and provided a biological basis for the results of epidemiologic studies. It also indicated an urgent need to control O_3_ pollution and to determine the pathogenic molecular mechanisms induced by exposure to O_3_.

## Data Availability

The original data presented in this study are included in the article/Supplementary Materials. Further inquiries can be directed to the corresponding authors.

## Supplementary Materials

The following supporting information can be downloaded at https://www.mdpi.com/article/10.3390/toxics13060468/s1, Table S1. The experimental plan in mice exposed to different doses of O_3_. Table S2. Changes of serum biochemistry parameters of mice exposed to O_3_ for 28 days. Table S3. Changes of hematology parameters for mice exposed to O_3_ for 28 days.
