# Supplementary material for: Subacute Inhalation Exposure of Mice to Ozone Induces Damage to Various Organs
Source: Toxics. 2025 May 31;13(6):468. doi: 10.3390/toxics13060468 (PMC12197127; doi:10.3390/toxics13060468)
Supplement: Supplementary file 1 [file toxics-13-00468-s001.zip › toxics-3621941-supplementary.pdf]

Supplemental Tables

Table S1. The experimental plan in mice exposed to different doses of O_3.

O_3 (ppm)	Number	Weight	Pulmonary function	Hematology and serum biochemistry	Extraction of BALF (the ligated side)	Histological analysis of the other side of lung	Histological analysis of other tissues	ELISA (frozen tissues)
0	1-6	✓	✓	✓	✓	✓	✓	
	7-12	✓	✓					✓
0.5	1-6	✓	✓	✓	✓	✓	✓	
	7-12	✓	✓					✓
1	1-6	✓	✓	✓	✓	✓	✓	
	7-12	✓	✓					✓
2	1-6	✓	✓	✓	✓	✓	✓	
	7-12	✓	✓					✓

Table S2. Changes of serum biochemistry parameters of mice exposed to O_3 for 28 days.

Clinical chemistry	O_3 (ppm)			
	0	0.5	1	2
TP (g/L)	56.2500±2.0137	56.1833±2.3953	57.0143±3.4334	54.6500±2.4858
ALB (g/L)	29.0000±0.6663	29.2500±0.9247	29.4857±1.4656	28.4167±1.1635
AST (U/L)	115.9500±15.5048	119.7000±20.9689	117.7000±28.1327	108.4667±13.2435
ALT (U/L)	26.9500±3.7082	25.3000±2.2909	25.5143±2.4155	23.5000±2.3057*
BUN (mmol/L)	11.4250±0.9221	10.7750±0.2556	9.6829±0.5512*	10.3583±1.7557
CREA (μmol/L)	24.6500±2.8062	23.3667±0.8914	22.9000±1.4154	20.6000±2.0377
GLU (mmol/L)	2.2150±0.5309	2.0717±0.5385	2.9514±0.6449*#	3.0100±0.2701*#
TG (mmol/L)	1.6117±0.4001	1.3367±0.3699	1.8329±0.2554#	2.2800±0.5315*#
CHOL (mmol/L)	2.4533±0.0995	2.4283±0.1437	2.3457±0.1698	2.4017±0.1492

The data were reported as means ± SD.

*, $P < 0.05$, compared with 0 ppm groups; #, $P < 0.05$, compared with 0.5 ppm groups.

Table S3. Changes of hematology parameters for mice exposed to O_3 for 28 days.

Hematology	O_3 (ppm)			
	0	0.5	1	2
WBC (10^9/L)	9.6583±1.4974	9.5133±1.5779	6.6217±1.3356*$^{\#}$	9.4533±1.3948$^{\&}$
RBC (10^{12}/L)	11.0467±0.5647	11.4067±0.2982	11.3350±0.4085	11.4650±0.1874
HGB (g/L)	160.5000±6.1237	163.5000±3.2094	162.3333±6.2503	166.8333±3.1885
HCT (%)	48.7833±2.1591	49.7167±1.0980	48.9000±1.9880	50.7667±0.7763
MCV (fL)	44.1833±0.6242	43.6000±0.2608*	43.1333±0.2503*	44.2667±0.5574$^{\#\&}$
MCH (pg)	14.5333±0.3141	14.3333±0.2338	14.3333±0.1862	14.5500±0.2074
MCHC (g/L)	329.1667±4.4460	328.8333±3.7639	332.1667±4.2151	328.6667±2.6583
PLT (10^9/L)	1257.0000±177.6041	1189.3333±334.6596	1328.1667±114.7614	1192.1667±77.7468
RDW-SD (fL)	21.9500±1.3605	21.2000±0.6812	20.3000±0.6841*	21.5000±1.1009
RDW-CV (%)	18.2667±0.3445	18.4333±0.6250	18.0000±0.4561	18.3167±0.3061
PDW (fL)	6.3667±0.1366	6.5667±0.1966*	6.3000±0.1414$^{\#}$	6.5500±0.1643$^{\&}$
MPV (fL)	6.2000±0.1789	6.2500±0.1975	6.1333±0.0817	6.1833±0.1169
PCT (%)	0.7367±0.0802	0.7433±0.1093	0.7550±0.0446	0.7467±0.0258
NEUT (10^9/L)	0.7050±0.1062	0.7350±0.1611	0.4617±0.1067*$^{\#}$	0.7417±0.2194$^{\&}$

Hematology	O_3 (ppm)			
	0	0.5	1	2
LYMPH (10^9/L)	8.7033±1.5265	8.4817±1.3884	6.0300±1.2665*#	8.4850±1.3479&
MONO (10^9/L)	0.1133±0.0197	0.1233±0.0308	0.0717±0.0232*#	0.1217±0.0449&
EO (10^9/L)	0.1267±0.0350	0.0517±0.0194	0.1633±0.2294*	0.0950±0.0367
BASO (10^9/L)	0.0100±0.0000	0.0100±0.0063	0.0067±0.0052	0.0100±0.0000

The data were reported as means ± SD.

*, $P < 0.05$, compared with 0 ppm groups; #, $P < 0.05$, compared with 0.5 ppm groups; &, $P < 0.05$, compared with 1 ppm groups.
